# The Influence of Design and Implementation Characteristics on the Use of Maternal Mobile Health Interventions in Kenya: Systematic Literature Review

**DOI:** 10.2196/22093

**Published:** 2022-01-27

**Authors:** Karen Sowon, Priscilla Maliwichi, Wallace Chigona

**Affiliations:** 1 University of Cape Town Department of Information Systems Cape Town South Africa; 2 Department of Computer Science and Information Technology Malawi University of Science and Technology Limbe Malawi

**Keywords:** human-technology interaction, maternal health, mHealth, mobile phone, utilization, Kenya

## Abstract

**Background:**

The growth of mobile technology in developing countries, coupled with pressing maternal health care challenges, has led to a widespread implementation of maternal mobile health (mHealth) innovations. However, reviews generating insights on how the characteristics of the interventions influence use are scarce.

**Objective:**

This study aims to review maternal mHealth interventions in Kenya to explore the influence of intervention design and implementation characteristics on use by maternal health clients. We also provide a starting inventory for maternal mHealth interventions in the country.

**Methods:**

Using a systematic approach, we retrieved a total of 1100 citations from both peer-reviewed and gray sources. Articles were screened on the basis of an inclusion and exclusion criterion, and the results synthesized by categorizing and characterizing the interventions presented in the articles. The first phase of the literature search was conducted between January and April 2019, and the second phase was conducted between April and June 2021.

**Results:**

A total of 16 articles were retrieved, comprising 13 maternal mHealth interventions. The study highlighted various mHealth design and implementation characteristics that may influence the use of these interventions.

**Conclusions:**

In addition to elaborating on insights that would be useful in the design and implementation of future interventions, this study contributes to a local inventory of maternal mHealth interventions that may be useful to researchers and implementers in mHealth. This study highlights the need for explanatory studies to elucidate maternal mHealth use, while complementing existing evidence on mHealth effectiveness.

## Introduction

### Background

The growth in mobile technology has led to the budding of mobile innovations such as mobile health (mHealth), whose use could solve some of the most persistent challenges in low- and middle-income countries. mHealth refers to “innovations that integrate the use of mobile and wireless devices to improve healthcare outcomes, healthcare services, and health research into care delivery” [1]. Infectious diseases and maternal health are the 2 main health outcomes where mHealth has had the greatest effect in developing countries [2]. This is no surprise because maternal health is one of the pressing needs in most resource-poor countries, with mortality rates being much higher in these countries, than in their high-income counterparts. Although maternal mortality in sub-Saharan Africa (SSA) dropped by 45% to 546 maternal deaths per 100,000 live births between 1990 and 2015, these figures are still much higher than those for European and Commonwealth of Independent States countries that were already at a figure of 69 maternal deaths per 100,000 live births in 2009, which had dropped by more than half to 25 maternal deaths per 100,000 live births by 2015 [3].

Pregnancy is a complex period in a woman’s life, and various factors influence the uptake and use of maternal health interventions to generate health outcomes. In low-resource countries, maternal health clients’ perceptions of care, quality of service, sociocultural, and socioeconomic factors may all contribute to how, why, and when maternal clients use interventions. Some studies have shown that maternal clients’ perceptions of health care providers may positively or negatively influence the uptake of services [4-6]. In addition, perceptions of quality of service and the level of satisfaction with quality of care may contribute to the motivation or delay in seeking care [4]. The price of services and the high direct and indirect costs of care may also impede access [7,8], although a woman’s autonomy in health care decision-making may also be a factor in her financial independence. Equally, uncertainties surrounding pregnancy from sociocultural beliefs [9,10] may influence how and when maternal clients interact with maternal services. Pregnancy in most parts of SSA is a largely collectivist experience. Other family members play a role in pregnancy-related decision-making and may influence a maternal clients’ use of maternal services [11,12].

Studies show that the “use of mobile technology can improve client knowledge base, service uptake and timely management of emerging pregnancy complications” [13], thereby improving maternal health outcomes. In information systems, however, it is well established that the characteristics of the technology and its context of use may influence its use in the first place [14-16]. Together with the sociocultural factors explored earlier, the interplay of factors to produce mHealth use outcomes proves complex. In a nascent field such as mHealth, whose evidence base is largely anecdotal, it is therefore useful to examine and understand the link between intervention characteristics and their use in light of contextual realities in which the interventions are implemented.

Although there have been many reviews of maternal and child health (MCH) mHealth interventions in low-and middle-income countries [17-22], most of these reviews have only studied the effectiveness of the interventions in terms of health and clinical indicators. One review [22] explored the influence of such interventions in MCH practices, such as clinic attendance and assisted delivery. Thus, to our knowledge, no review has explored the interventions' design and implementation characteristics in light of their use.

### Objectives

This review seeks to contribute to exploring the influence of design and implementation characteristics on the use of MCH mHealth interventions. Unlike most reviews, we opt to adopt a country-specific analysis to allow for depth rather than breadth of analyzing mHealth interventions. Therefore, we chose a country that has a high number of implemented mHealth programs as a case, because insights from such a country may be beneficial in charting a direction for mHealth in SSA. Kenya is one of the countries whose maternal mortality is still high, ranking 19th in both SSA and the world. Owing to its concomitant growth in mobile technologies, Kenya has become a hot spot for mHealth interventions [23,24]. Mobile growth statistics show that, together with South Africa and Nigeria, Kenya’s mobile economy ranks high in Africa [25]. Mobile phone ownership has grown exponentially over the past decade from 33% in 2007 to an estimated 86% in 2018 [26], with over 100% penetration rate in 2020, attributed to multiple SIM card ownership [27]. Our interest is in maternal health interventions with which the maternal health clients directly interact, rather than those delivered via a health care worker or volunteer. Hence, the high penetration of mobile phones—that provide a channel over which these mHealth interventions are delivered—made Kenya an interesting case to explore the objectives of this study.

Governments have identified the need for inventories of mHealth programs as an important prerequisite for tracking eHealth innovations in countries [28]. In many countries, the lack of clarity on what maternal mHealth interventions exist could potentially further *pilotitis* and duplication of efforts among implementers. Consequently, in addition to allowing for depth in tracing interventions, a tighter geographic focus allows for the study to contribute to developing a country-specific preliminary inventory. Although Njoroge et al [29] conducted a review in Kenya, their review was not targeted specifically at maternal health; therefore, it may not offer such an inventory of maternal mHealth interventions.

We believe that this review will complement existing studies that highlight the influence of mHealth use on MCH practices and outcomes by elucidating how the characteristics of such technologies may influence use. We think that this is important because only by their successful use will mHealth interventions achieve their lauded potential to improve maternal health in developing countries. These insights would be useful for mHealth designers and implementers and provide a direction for areas that need to be strengthened in mHealth research. The resulting inventory may also be useful to maternal mHealth implementers and the government to consider existing interventions before implementing new ones, in a bid to promote collaboration around mHealth solutions, and decrease *pilotitis*.

## Methods

### Overview

This study adopted a systematic review to rigorously identify and select maternal mHealth interventions to be analyzed. Many eHealth implementations in Kenya have not been reported in peer-reviewed literature [29]. Therefore, the study adopted a combination of sources to capture both peer-reviewed and gray literature. Table 1 summarizes the search strategies used. The combination of search terms from Textbox 1 to form search phrases consisted of 2 to 3 components: a word that described mHealth and related technology, a word that described maternal health and pregnancy, and the country name, that is *Kenya*, to limit the results to our geographical area of interest. We conducted the first phase of the literature search between February and April 2019 and the second phase between April and May 2021. We have used the terms intervention and program interchangeably to describe a specific mHealth project.

**Table 1:** Search strategy (adapted from Njoroge et al [29]).

Step	Peer-reviewed sources	Non–peer-reviewed sources
1	Peer-reviewed sources from the databases EBSCOhost, PubMed, Scopus, Web of Science, ACM, and Google Scholar	Non–peer-reviewed sources, such as web-based portals for Kenya’s most read newspapers (Nation and Standard) and organizational reports (WHO^a^, mHealth^b^ Alliance, and IDRC^c^)
2	Manual searches of references in documents	Web portals for eHealth projects in Kenya
3	N/A^d^	Profit-based and nonprofit organizational websites
4	N/A	Personal communication with players

^a^WHO: World Health Organization.

^b^mHealth: mobile health.

^c^IDRC: International Development Research Centre.

^d^N/A: not applicable.

**Keywords used for the systematic literature review.**

**Keywords**

- Kenya, mhealth and/or m-health, mobile health, maternal, maternal, neonatal and child health and/or MNCH, pregnant wom?n, pregnan*, mobile, mobile phone, mobile telephon*, innovation*, cell phone, text messag*, SMS, voice call*

### Search Strategy

In our first round of searching for peer-reviewed sources, we selected literature sources, databases, websites, and registers based on their relevance and likely coverage of literature and applied the search strategy detailed in Table 1. The databases for peer-reviewed sources included EBSCOhost (capturing resources from Academic Search Premier, CINAHL, LISTA, MEDLINE, Newspaper Source, and SocINDEX), PubMed, Scopus, Web of Science, Association for Computing Machinery, and Google Scholar. Following this, we conducted a manual search using reference trailing to augment and fill in any gaps in our search strategy.

The primary author (KS) developed the search terms by reviewing previously published peer-reviewed studies. The search terms were reviewed and tested for completeness by the second author (PM). We used the same terms in both peer- and nonpeer-reviewed searches. The search terms included Boolean-paired key words, variants, and spelling variations as detailed in Textbox 1.

Our second round of search was targeted at gray sources to identify interventions that were existent but which might not have been formally evaluated. The non–peer-reviewed sources incorporated web-based portals for eHealth, profit-based and nonprofit organizational websites, newspaper articles, organization blogs, and reports. The final step, which can be deemed rather subjective, was initiated by the primary researcher through personal communication with mHealth players in Kenya, linked to the interventions retrieved from gray sources. This was done to gather missing information and validate what had been accessed from the websites, as well as to trace other programs that the researchers may have missed. To start with, the researcher contacted 2 people, who provided referrals to 2 other people, bringing the total number to 4 (Textbox 2). Interviews were conducted in person. The participants offered some high-level details of the programs, most of which had already been gathered from their websites and publicly available resources.

**Participants and their affiliations.**

**Participants and affiliations**

- Participant 1 was affiliated with BabyMed.
- Participant 2 was affiliated with TotoHealth.
- Participant 3 was affiliated with Amref, Kenya.
- Participant 4 was affiliated with Amref, Kenya.

### Inclusion and Exclusion Criteria

Eligible materials included journal articles, conference proceedings, and published information from governments and other organizations’ portals. Peer-reviewed sources were required to have the full text available on the web for review. Gray references to interventions were included if the existence of the intervention could be established by more than 1 source or personal communication with key players or both. As reflected in government reports and documents, English is the business language in Kenya. Having confirmed that there was a corresponding English source for the few Swahili sources that we could identify, we chose to include articles that were published in English. We did not apply any year restrictions to the search because mHealth is fairly nascent in most low-resource countries.

As a guide for the selection of maternal mHealth programs, we adopted the World Health Organization’s definition of maternal health as the health of women during pregnancy, childbirth, and 6 weeks post partum. Therefore, in general, programs that addressed other areas in the reproductive, maternal, neonatal, and child health continuum were included only if they had a maternal health component delivered to maternal clients during this period. In the same manner, as HIV contributes to approximately 20% of maternal deaths [30], we included prevention of mother-to-child transmission and antiretroviral treatment adherence programs, which are initiated during pregnancy and targeted at improving pregnancy outcomes for maternal clients.

Articles were included only if the mHealth interventions they discussed were immediately and directly related to the improvement of maternal health outcomes. In addition, the peer-reviewed citations needed to have some evaluation information regarding the requisite interventions. Protocol-study dyads were included to provide a rich description of the intervention. For programs identified from gray sources, the inclusion depended on a verifiable existence, which was done by double-checking with other sources, typically by entering the intervention name as a search text on Google Search (Google, LLC) or by talking to health players (Table 1 and Textbox 2).

Articles on mHealth programs that did not have evidence of maternal outcomes were excluded, and so were those that lacked evidence of outcomes in Kenya. We also excluded literature reviews and studies whose main objective was to describe the development of mHealth system prototypes, without having an actual (not beta) deployment where maternal clients interacted with it. Study protocols whose evaluation outcomes could not be traced were excluded because they lacked findings on which user experiences could be assessed. Articles describing interventions that were purely supply facing were also omitted, as client experiences cannot be best explained by observing supply-side use. mHealth programs were counted only once if they were discussed in more than 1 article.

### Screening Process

**Figure 1:**
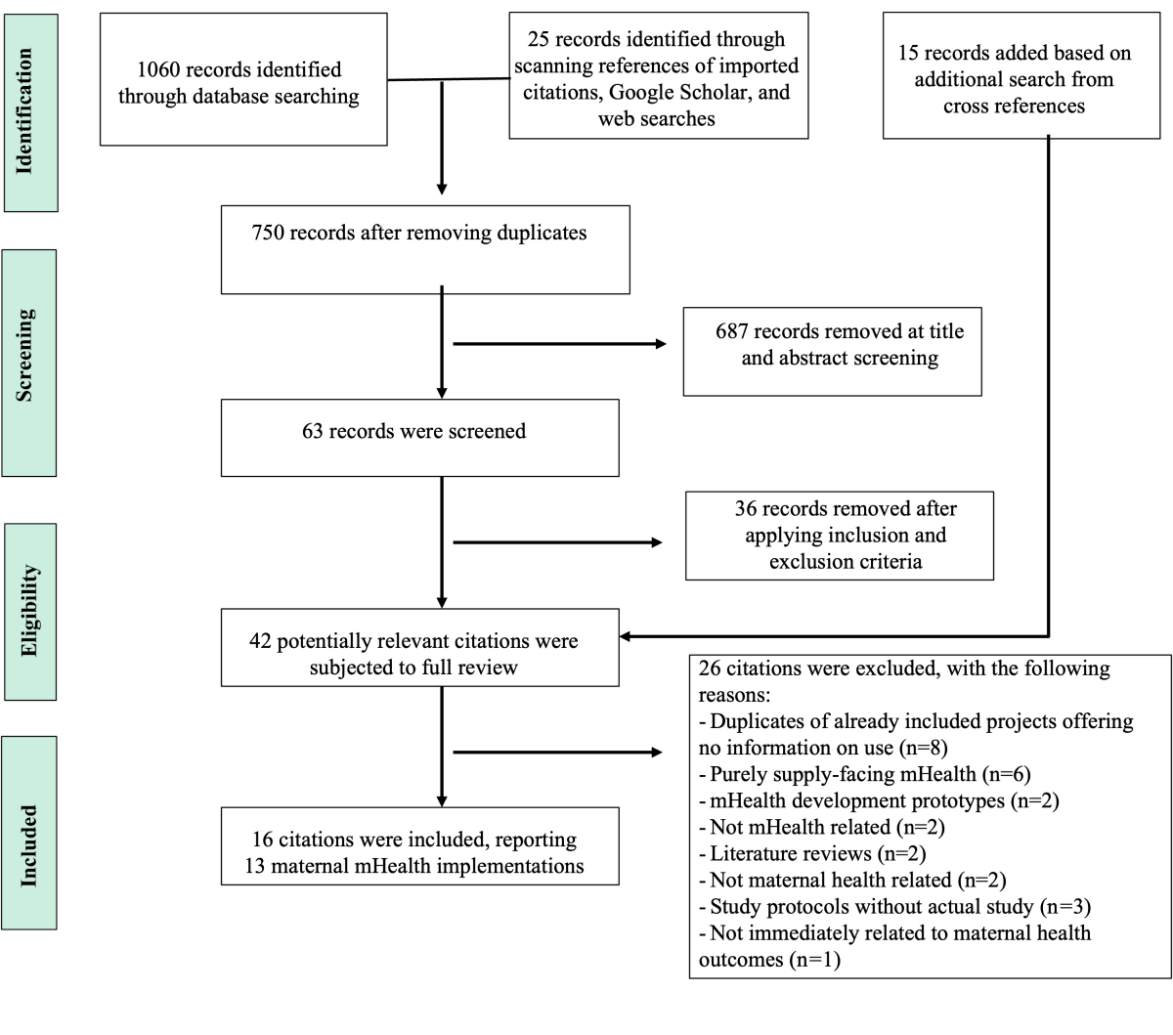
Literature screening process. mHealth: mobile health.

The primary author (KS) applied the search terms to the search sources, imported results to EndNote (Clarivate, Inc), removed duplicates, and screened for inclusion based on title and abstract, and then by skimming through the full article. To determine the final inclusion, 2 authors independently reviewed the full text of the potential citations. At this stage, citations that were supply facing, such as those targeted for use by community health workers or volunteers, were excluded. The screening process is shown in Figure 1.

### Synthesis of Results

Using the search strategy detailed in Table 1, we identified 1085 citations from peer-reviewed and non–peer-reviewed sources. An additional 15 citations were retrieved through additional searches (Figure 1). After full review, a total of 16 citations were included, featuring 13 unique client-facing maternal mHealth project interventions (Tables 2 and 3).

**Table 2:** Aggregated characteristics of the maternal mobile health interventions in Kenya (N=13).

Implementation characteristics		Technology used, n (%)			
		SMS	SMS + voice	Other	Total
**Other stakeholders^a^**					
	Health care providers	7 (54)	3 (23)	1 (8)	11 (85)
	Others	4 (31)	0 (0)	1 (8)	5 (38)
**Application area**					
	Education and behavior change	9 (69)	2 (15)	1 (8)	12 (92)
	Others	0 (0)	1 (8)	0 (0)	1 (8)
**Location**					
	Urban or periurban	4 (31)	2 (15)	1 (8)	7 (54)
	Rural	4 (31)	1 (8)	0 (0)	5 (38)
	Both	1 (8)	0 (0)	0 (0)	1 (8)

^a^Three interventions involved both health care providers and other stakeholders and were thus tallied twice.

**Table 3:** Design and implementation characteristics of maternal mobile health interventions in Kenya.

Author	Title	Intervention name	Design and implementation characteristics	Outcomes related to use
Perrier et al [31]	Male Partner Engagement in Family Planning SMS Conversations at Kenyan Health Clinics	Unnamed SMS platform	Toll-free SMS Automated weekly messages Messages to participants with enrolled partner using inclusive wording A question related to the message topic to encourage engagement Home visits to reach the partners Dedicated staff to answer the messages Privacy in group messaging feature by sending messages separately to each person’s individual phone among couple dyads	Including the male partner engaged more households than would otherwise be included in the conversation Use significantly dropped when intervention stopped being free Individualized responses from study staff help build a level of trust in the SMS system opening the door to more engagement Privacy within couple dyads encouraged conversation
Perrier et al [32]	Engaging Pregnant Women in Kenya with a Hybrid Computer-Human SMS Communication System	Mobile WACh	Two-way SMS Human-mediated (computer automates bulk-sending of messages and responses are tailored by a staff) Allow unstructured messages Send personalized time-sensitive messages Each message salutes with mother's name Toll-free Dedicated nurse tells mother about intervention and enroll her, highlighting that the intervention is free Language choices according to user A question related to the message topic to encourage engagement.	Unstructured messages increase access by allowing users with little experience to participate and engage Stage personalized messages made women to feel cared for The availability of a nurse to answer questions made women feel cared for
Ronen et al [33] and Drake et al [34]^a^	SMS messaging to improve ART^b^ adherence: perspectives of pregnant HIV-infected women in Kenya on HIV-related message content	Mobile WAChX	Tailored messages based on woman’s stage in the pregnancy or postpartum continuum SMS delivered in preferred language Message includes question related to the message topic that solicits engagement Women in two-way arm communicate with the study nurse via SMS at any time SMS content developed in consultation with target group Congratulatory message sent when ANC visit is attended Option to opt out at any point Messages include salutation with nurse’s and client’s name	Messages helped women feel cared for Messages improved perceptions of care Concerns about confidentiality in receiving HIV-overt content (mainly because of third-party access to their phone) Anonymity in medium (SMS) resulted in patients feeling that they could send overt HIV messages to the nurse
Fairbanks [35]	Perceptions of SMS content for Pregnant and Postpartum Kenyan Women Infected with HIV	Mobile WACh-X	Tailored SMS Confidentiality (sending covert rather than explicit messages)	Feeling cared for and supported Improved engagement in HIV and MCH^c^ health outcomes Caring messages improve provider-patient relationships Messages serve as a catalyst to engaging in conversation with their partners
Harrington et al [36]	An mHealth^d^ SMS intervention on Postpartum Contraceptive Use Among Women and Couples in Kenya: A Randomized Controlled Trial	Mobile WACh XY	Question at the end of message designed to promote SMS dialogue Message content corresponding to participants’ gestational age or postpartum week Semiautomated messages with nurse’s input to tailor responses to client questions Female participants recruited by female nurses and male by male study staff Toll-free	Two-way SMS with a nurse, and an involved partner increased postpartum contraceptive use. Two-way SMS results in a high level of participant engagement in SMS dialogue with study nurses
Pintye et al [37]	Two-Way Short Message Service (SMS) Communication May Increase Pre-Exposure Prophylaxis Continuation and Adherence Among Pregnant and Postpartum Women in Kenya	mWACh-PrEP	Two-way SMS to allow real-time communication A dedicated nurse to receive and respond to messages SMS message development informed by theory Toll-free SMS service Messages include salutation with nurse’s and client’s name Messages ends with a question to solicit engagement Autonomously exit the program Multiple language options	Real-time communication facilitated continued pre-exposure prophylaxis use High SMS engagement from participants in response to automated push messages Women reported consulting by SMS with the nurse and continuing pre-exposure prophylaxis because of the nurse’s advice Diminished response to automated messages after one month
Patel et al [38]	Providing Support to Pregnant Women and New Mothers through Moderated WhatsApp Groups: a Feasibility Study	Jacaranda Health	Moderator's participation in the service was part-time Skilled moderator (with basic nursing background) asks questions to stimulate conversation 10 women/group with similar gestational age Moderator referred participants to the health facility to address individual medical questions (maintaining patient confidentiality)	Groups created small community for women to learn from and support each other Dissatisfaction over delayed responses from the nurse that resulted in some maternal clients abandoning the intervention
Bardosh et al [39] and Awiti et al [40]^e^	Operationalizing mHealth to improve patient care: a qualitative implementation science evaluation of the WelTel texting intervention in Canada and Kenya	WelTel Kenya-2 Grand Challenges Canada	Interactive two-way SMS with optional voice call from provider to patient Manual messaging Free of charge Occasional push messages	Two-way SMS allowed patients to seek feedback on questions and problems, giving the sense that someone cared The two-way communication Improved relationship between patients and providers 20% of HIV patients enrolled in the intervention immediately; 80% enrolled only after being encouraged by other patients High number of nonrespondents that did not respond to the weekly messages
Fedha [13]	Impact of Mobile Telephone on Maternal Health Service Care: A Case of Njoro Division	Njoro Hospital	Optional provider-patient follow-up	No data on user experiences (evaluation done based on health outcome indicators only)
Finocchario-Kessler et al [41]	A Pilot Study to Evaluate the Impact of the HIV Infant Tracking System (HITSystem 2.0) on Priority PMTCT^f^ Outcomes	HITSystem v2.0)	Participants choose preferred message content and frequency All message content in one language—Kiswahili	Standard care participants more likely to be disengaged from care than those receiving intervention care Higher preference for daily SMS than weekly or monthly
Jones et al [42]	A Short Message Service (SMS) increases postpartum care-seeking behavior and uptake of family planning of mothers in peri-urban public facilities in Kenya	PROMPTS	Messages sent in Swahili to lower accessibility barriers SMSs were free of charge Stage-relevant messages Messaging around postpartum checkups was broad, and explain why postpartum care was imperative	Women who were told by the health care provider to come back for a postpartum checkup were 14 times more likely to come back than those who received messages only
Germann et al [43]	Jamii Smart| KimMNCHip–referrals, mSavings and eVouchers	KimMNCHip (Jamii Smart)	Mobile wallet facilities allow mothers to save and manage their delivery and health care costs	No data
BabyMed [44]	BabyMed	Baby Med	Time-sensitive reminders sent to mothers and partners One message per week delivered to husbands and other family members to support the mother and baby	Received positive feedback in involving partners
Luseka et al [45]	An Evaluation of Toto-health Mobile Phone Platform on Maternal and Child Health Care in Kenya	Toto Health	Time-sensitive and targeted information and reminders sent to mothers and their partners Initially toll-free and later made a paid for service Inconsistency in timing of messages Periodic visit by a Toto health personnel to the mother	Feeling cared for Partners felt that they had a friend to educate them about what’s happening to their wives Abandoned used when intervention was introduced as a paid for service Dissatisfaction with the inconsistency Increased trust on the system whenever personnel visited the mothers

^a^The research protocol for Ronen et al [33].

^b^ART: antiretroviral therapy.

^c^MCH: maternal and child health.

^d^mHealth: mobile health.

^e^The research protocol for Bardosh et al [39].

^f^PMTCT: prevention of mother-to-child transmission.

We sought to characterize the interventions according to the technology channel in use, the involvement of other stakeholders, mHealth application area as documented in Labrique et al [46], and location of implementation, whether urban or rural (Table 2). Where an implementation could have been placed in more mHealth application areas, or was not sufficiently described to understand its content, it was placed in the category in which the researchers deemed as the best fit.

In synthesizing the articles, we focused on the design and implementation characteristics and the impact of these on the use experiences of users, as described in the citations. We also took note of the type of evaluation, for example, if it was a randomized controlled trial (RCT) or another type of evaluation.

## Results

### Intervention Characteristics

Table 2 describes the aggregated characteristics of the identified mHealth programs, whereas Table 3 elaborates on their design, implementation, and use details.

#### Use of SMS in mHealth

SMS was the predominant technology used in both urban and rural maternal mHealth implementations. Some of the most common uses of SMS include the delivery of health information and appointment reminders. Interventions implemented different SMS calibrations, including one-way (push) or two-way SMS, most of which were computer-automated, with a human component to respond to client questions, often referred to as hybrid systems [31-33,36,37,41,42]. Fewer programs incorporated other channels such as voice [13,39], which were mostly available for the health care provider for follow-up purposes, and even fewer reported using other messaging options such as WhatsApp (Meta Platforms, Inc) [38].

#### Target Users and mHealth Application Areas

As expected, all interventions mainly targeted the maternal client with the aim of client education and behavior change. Most interventions also involved the health care provider to either follow up on clients or to respond to client questions [13,32,33,37,39]. In addition to involving health care workers, few interventions also involved other stakeholders in the women’s life such as their partners [33,36] and other women to offer group support [38].

#### Implementation Location

One of the main motivations for the use of technologies such as mHealth by health programs is to extend the geographic reach of health care, particularly in resource-strained environments [47]. This may be in the form of addressing the shortage of health care providers, as well as unequal distribution of health services that may exist between social groups such as urban and rural or rich and poor. The results of this review suggest that a higher number of interventions were piloted in urban and periurban areas.

#### Nature of Programs and Evaluation

Most of the deployments were short-lived funded pilot studies, whereas 2 interventions [44,45] represented proprietary social enterprises that were privately owned. Almost all the interventions that had been evaluated were RCTs [32,33,36,37,42]. Of the 3 interventions whose evaluation details could not be traced, 2 (66%) were privately owned social enterprises, and one was a multi-stakeholder program [43].

### Influence of Design and Implementation Characteristics on Use

#### Engaging Other Stakeholders May Promote Use

Many interventions involve health care providers in the implementation process to execute various roles. For example, in the Mobile WACh program [32], a nurse was assigned to tell the maternal clients about the intervention, enroll them, and highlight that the intervention was free. Most interventions that implemented two-way SMS used a dedicated health care provider to respond to maternal health client queries via SMS [31-33,37,39]. In rare cases, such as for Jacaranda Health, for implementing a moderated WhatsApp group support system, the intervention made use of part-time staff [38]. These interventions, which were integrated into mainstream care by involving health care providers at the local health facilities that the maternal client attended, resulted in improved perceptions of care and better provider-patient relationships. Fewer interventions that engaged other community members such as male partners, for example [31,45], showed that mHealth educational messages led to better health outcomes, resulting from increased engagement with the mHealth content.

#### Design and Implementation Characteristics May Facilitate Use

The results of our synthesis show that interventions were more readily adopted and used when they were offered free of charge [31,32,36,37,39,42]. Interventions whose content was not stage-based, such as mWACh-PrEP, experienced diminished use after some time. The diminished use was likely experienced when the users felt sufficiently onboarded regarding the logistics and continuation or discontinuation of pre-exposure prophylaxis [37]. However, when interventions delivered timely, useful, time-sensitive, stage-based information, accompanied by an appropriate *tone* and *voice*, the mothers felt *cared for* [32,33,35,39,45] and continued active use. The additional access to a health care provider to answer questions [31-33,37] resulted in the women developing positive perception toward care and toward health care providers.

The anonymity offered by SMS, as well as the anonymity in message content, especially where the target users were HIV-infected women, influenced the way maternal health clients interacted with the intervention. In Mobile WAChX [33], maternal health clients expressed concerns about confidentiality in receiving HIV-overt content, mainly because of possible third-party access to their phones. The anonymity of the SMS channel, compared with face-to-face communication, also afforded users the opportunity to engage with overt questions. In Mobile WACh, Mobile WAChX, and Mobile WACh-PrEP [32,33,37], the messages included a salutation to the maternal client using her name before the actual message content. This was also seen to improve the perception of personalized care among the maternal health clients, which further resulted in them feeling *cared for*.

#### Frugal Technology Such as SMS Promotes Opportunities for Use

Almost all interventions reported the use of SMS to deliver messages related to maternal health care. mHealth programs, particularly those using SMS, have been shown to increase the uptake of maternal health services in developing countries [32,36,42]. In the randomized trials, the users in the two-way SMS intervention arms showed better engagement with the mHealth intervention [31-33,36,37,39]. As these were implemented free of charge, maternal health clients were able to address health-related concerns by sending a message to the health care provider and receiving feedback in real time. Unstructured message implementations also allowed increased access and use by allowing users with little technical experience to participate and engage with the interventions [32].

## Discussion

### Principal Findings

The study’s findings suggest that various design and implementation characteristics may influence use. From our analysis, we identified three main considerations: (1) engaging other relevant stakeholders to promote use, (2) designing interventions with characteristics that facilitate and promote use, and (3) considerations for the use of SMS technology.

### Engaging the Maternal Community of Purpose in the Design Section and Implementation

Various individuals ranging from health care providers to other community members share a common interest and have stakes in women’s pregnancies in most low- and middle-income countries. These stakeholders form a *community of purpose*. Involving health care providers in the implementation process may have positive outcomes regarding the use of mHealth interventions. Health care providers wield power in many health contexts, especially when they are regarded as gatekeepers of medical information. A similar observation was made in the literature, suggesting that health information technologies are likely to be more successful if providers encourage patients to use them [48]. Other findings in technology acceptance literature support that people may adopt technology based on the belief that important others think that they should do so [49,50].

Having a *human face*, such as a physician, who in the implementation context represents a trusted entity [51], could also promote adoption by minimizing the perceived risks and uncertainties about using the intervention, like clarifying the toll-free access [32]. Furthermore, having a trusted human face to introduce the intervention has been reported to avert concerns about perceived risks such as airtime loss that may prevent adoption [16], a concern that might be more pertinent in lower-income user groups. As health care providers are also considered a trustworthy source of care [51], their involvement in interventions may help legitimize mHealth information, thus averting the maternal clients’ perceived risks brought about by cultural beliefs related to certain maternal care practices and habits. In support of these arguments, adoption theories have suggested that mass media alone is not enough to drive the adoption of technology [14]. Therefore, they point to the need for rich channels of communication (eg, face-to-face communication) to share information about new technology in contexts where the personal and sociocultural characteristics of the target users result in high uncertainty regarding technology. Maternal health in developing countries represents one such context, where the uncertainty in using technology may additionally be attributed to the overall uncertainties surrounding the pregnancy experience.

In addition to health care providers, various individuals share a common interest and have stakes in women’s pregnancies in developing countries. Although older female relatives provide care and support, male partners are often responsible for the financial needs of the maternal clients [5,11]. During pregnancy, women especially rely on family support for responsibilities related to childcare and other areas that are considered female domains [52]. Therefore, being away from family significantly reduces family support for women [53]. The increased need for support may, therefore, promote the success of novel interventions where maternal health clients are brought together to offer group support with the direction of a trained health care provider, as seen in the study by Patel et al [38].

Engaging stakeholders such as partners may increase engagement with mHealth content because of the interdependent nature of the maternal health care–seeking context. An intervention that includes partners and other significant others in the maternal health client’s life may serve to reduce the negotiation that the maternal health client must engage in to ensure her use if the intervention is a culturally appropriate behavior [51]. Rogers et al [14], in the diffusion of innovations theory, uses the term compatibility to refer to the degree to which using an innovation is perceived as consistent with the existing sociocultural values and beliefs of the adopters.

Better health outcomes may also reflect the affordance that technology offers to negotiate cultural *rules*. For example, although pregnancy is often considered a woman’s domain in which men are not involved [54,55], designing interventions that involve men engenders more of their participation without causing overt disharmony in social norms. Altogether, engaging the relevant stakeholders in the design and implementation process could have positive outcomes on mHealth use because of the interdependent nature of the maternal health care–seeking context in developing countries, especially in societies that are more collectivist in nature. However, interventions also need to be aware of the complex interpersonal relationship dynamics in a maternal health context [31] when calibrating the community of purpose engagement.

### Designing Interventions With Characteristics to Facilitate Use

Although the success of toll-free interventions could be linked to the socioeconomic status of maternal clients, toll-free services may also have increased the trialability of the intervention, as observed by Sowon and Chigona [16]. The trialability of an innovation is positively correlated with the likelihood of its adoption [14]. In health care, trialability is often linked to minimal financial investment [56].

The findings also suggest that the quality of mHealth information and what it evokes in users is crucial to maternal mHealth. Some researchers have suggested that when technology is *faceless*, users build trust by assessing the quality of the information [57], which is often used by mHealth users as a proxy for quality of service, especially in innovations such as mHealth, where health information is critical. Other studies have observed that mHealth may be underused when its users express low trust in their integrity and benevolence or when there is no demonstration of in-depth knowledge and clear concise information [58,59]. The additional access to health care providers provided in two-way SMS calibrations may further increase the perceptions of usefulness, thus engendering use. Subsequent responses from health care providers build positive perceptions toward the providers. Altogether, the quality of the information, perceived usefulness, and positive attitude toward providers could result in positive perceptions of care.

Other characteristics such as anonymity may positively influence use because they afford users the opportunity to engage matters of stigma or cultural taboos associated with certain conversations [60,61]. Though tailoring messages with a client’s name may promote personalization, it may also thwart the potential benefits to be gained from perceived anonymity. However, because the findings show that there are other options to personalization, such as sending time-sensitive messages based on a woman’s stage of pregnancy, the decision on what to personalize in an intervention should be context-dependent. Interventions need to identify the most salient characteristics to be tailored for the delivery of health interventions [62]. As seen in the results, personalization is important because such characteristics influence the clients’ perceptions of quality of service, and subsequently, their judgments on satisfaction and use. Satisfaction is necessary for the continued use of mHealth interventions [63].

### Opportunities and Challenges With SMS

Our results confirm the findings in other reviews [17,21,29], indicating that most mHealth interventions in developing countries show a proliferation of interventions that uses short messaging. The popularity of SMS may be ascribed to the fact that it is accessible even on the most basic feature phones and attracts lower costs than, for example, voice calls. Most rural and underserved populations are likely be in ownership of a feature phone and not a smartphone [26]. In addition, SMS is easy to use because it does not require high literacy levels. Although the reasons for SMS popularity may be sensible, other features that allow group interaction, as demonstrated by Patel et al [38], may provide more novel solutions, especially in situations where maternal clients are already separated from their usual family support, such as in urban areas. Pure SMS interventions may also exclude those who cannot read and write, thus creating further gaps in health.

Although push messages may be less complicated for mHealth providers and designers to offer [42], they lack the robustness and flexibility that two-way SMS offers for users. One-way SMSs that allow users to submit text to a server lack a feedback loop that leaves a user wondering if their message was received [64]. On the contrary, two-way SMS interventions allow consumers to engage with care and engender better use. Some of the programs reviewed in this study reported innovative ways of engaging users in SMS. For example, posing reflective questions with most messages to solicit engagement [31-33], was seen to have positive outcomes on how maternal clients engaged with the intervention.

These findings suggest that unstructured message formats increase usability. However, such programs require human intervention because the automation of responses would be complex. Using humans to respond to client questions may create further bottlenecks, which may create dissatisfaction and limit use, as seen in the study by Patel et al [38]. In their study, maternal health clients abandoned the intervention because they were dissatisfied with the delayed responses. The period of waiting for care may have negative implications on health outcomes, as maternal health clients may engage with alternative sources of care [5]. These alternative sources may offer contradictory information to mainstream care, thus worsening health conditions. Hence, finding ways to engage users, especially given the asynchronous nature of SMS, will be critical to the long-term success of such interventions.

### Conclusions and Recommendations

This review intended to provide insights on how mHealth design and implementation characteristics may influence use by reviewing and analyzing maternal mHealth interventions in Kenya. The 2016-2030 Kenya National eHealth Policy also identifies the need for mHealth inventories as a prerequisite to managing the licensing and audit of interventions by the Ministry of Health. Thus, the results of this study offer a potential maternal mHealth inventory.

The findings reveal that mHealth design and implementation characteristics play a critical role in how maternal health clients use mHealth interventions. Certain characteristics could promote the use of mHealth interventions but the causal relationship largely depends on the context, as users interact with technology within their local realities. The study identified that involving stakeholders, having characteristics that facilitate use, and how SMS is deployed in interventions are all factors that could influence use.

However, these insights are generated from evaluations that only marginally discuss experiences of use. This review reveals that most mHealth evaluations [13,32,33,40,65-68] are implemented as RCTs, which mostly evaluate maternal health interventions based on quantitative health outcome indicators. Thus, there is little evidence of studies explaining the mechanisms, that is, why, when, and how interventions work or do not work. This calls for researchers and implementers to conduct more research in this area, to understand how mHealth interventions generate outcomes, or how they are used in their relevant contexts. One way to do this is to theoretically elaborate on the findings of this study to explain the mechanisms by which the design and implementation factors produce varied mHealth use outcomes. Such studies guided by theory will make it more possible to generalize results beyond a specific context, which may help in understanding how and whether to scale interventions. Although RCTs will remain useful in assessing the effectiveness of mHealth, they will be insufficient if adopted as the only method [69]. Qualitative investigations, especially on use, will complement RCTs and provide better evidence for mHealth.

### Limitations

This study had some limitations. Some of the data derived from the articles included in this study were from evaluations of mHealth interventions that did not purposefully report user experiences of the interventions. Consequently, the data may be insufficient for generalization. In addition, it proved difficult to reach the players to interview them. We believe that there may be other small-scale interventions that could have been implemented, which could have been identified only by the stakeholders involved in their implementation. As we depended largely on publicly available resources, the list of maternal mHealth implementations presented here may not be complete, and the findings are also limited to what could be accessed.

## Abbreviations

MCH: maternal and child health
mHealth: mobile health
RCT: randomized controlled trial
SSA: sub-Saharan Africa
